# Supplementary material for: Room-temperature quantum emission from interface excitons in mixed-dimensional heterostructures
Source: Nat Commun. 2024 Apr 11;15:2871. doi: 10.1038/s41467-024-47099-6 (PMC11009238; doi:10.1038/s41467-024-47099-6)
Supplement: Supplementary file 1 — Supplementary Information [file 41467_2024_47099_MOESM1_ESM.pdf]

Supplementary Information

Room-temperature quantum emission from interface excitons in mixed-dimensional heterostructures

Nan Fang[1,*], Yih-Ren Chang[1], Shun Fujii[2,3], Daiki Yamashita[2,4], Mina Maruyama[5], Yanlin Gao[5], Chee Fai Fong[1], Daichi Kozawa[1,2,6], Keigo Otsuka[1,7], Kosuke Nagashio[8], Susumu Okada[5], Yuichiro K. Kato[1,2,*]

[1]Nanoscale Quantum Photonics Laboratory, RIKEN Cluster for Pioneering Research, Saitama 351-0198, Japan

[2]Quantum Optoelectronics Research Team, RIKEN Center for Advanced Photonics, Saitama 351-0198, Japan

[3]Department of Physics, Keio University, Yokohama 223-8522, Japan

[4]Platform Photonics Research Center, National Institute of Advanced Industrial Science and Technology (AIST), Ibaraki 305-8568, Japan

[5]Department of Physics, University of Tsukuba, Ibaraki 305-8571, Japan

[6]Research Center for Materials Nanoarchitectonics, National Institute for Materials Science, Ibaraki 305-0044, Japan

[7]Department of Mechanical Engineering, The University of Tokyo, Tokyo 113-8656, Japan

[8]Department of Materials Engineering, The University of Tokyo, Tokyo 113-8656, Japan

* email: nan.fang@riken.jp, yuichiro.kato@riken.jp

Supplementary Note 1:
Absence of defect emission from pristine suspended CNTs

We illustrate several PL spectra in Supplementary Fig. 1 from pristine suspended CNTs before formation of heterostructures. All these tubes show a main peak from the E_{11} state with high PL intensities. Weak peaks at ~0.14 eV below the E_{11} states denoted as E_K are the K-momentum states [1-3]. Besides these E_{11} and E_K peaks, no other peaks related with defects could be resolved.

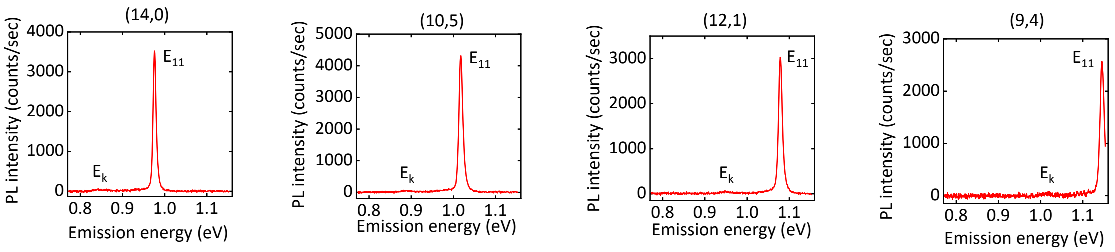

Supplementary Fig. 1 | PL spectra from pristine CNTs. The CNTs shown here are used for the formation of the heterostructures in Fig. 2. The excitation energy is adjusted to E_{22} and the power is 10 μW.

Supplementary Note 2:

Layer number dependence of PL from suspended WSe_2 flakes

The PL from the suspended WSe_2 flakes displays two primary peaks that vary with the layer number, which are assigned to A exciton and indirect exciton (IDX). As illustrated in Supplementary Fig. 2, the peak energy of IDX is more sensitive to the layer number than that of the A exciton, which is consistent with previous studies [4]. It is worth noting that the PL emission from the WSe_2 flakes is at a significantly higher energy than the newly observed IX peaks.

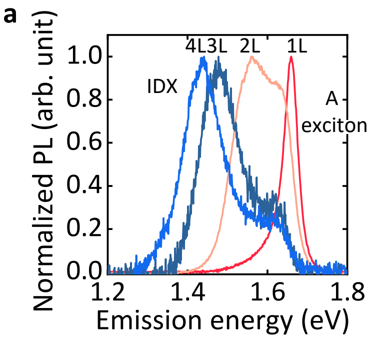

Supplementary Fig. 2 | Layer-number dependent PL spectra from the suspended WSe_2 flakes. a, PL spectra from the suspended WSe_2 flakes with different layer numbers. The excitation power is 10 μW. **b**, The extracted A exciton and IDX peak energies as a function of the WSe_2 layer number.

Supplementary Note 3:
Temporal evolution of PL in a CNT/WSe_2 heterostructure

The PL emission showcases a significant temporal evolution before the stable interface excitons form. Supplementary Fig. 3a illustrates a time trace of PL from a freshly prepared (9,4) CNT/2L WSe_2 sample. Two peaks, E_{11} (1.112 eV) and E_K (0.975 eV) are clearly observed, where E_K corresponds to the K-momentum exciton. In addition, new peaks sporadically appear at various energies, which are attributed to interface excitons. The representative PL spectra are shown in Supplementary Fig. 3b. The eight IXs, located at energies of 0.825, 1.069, 0.881, 1.006, 0.964, 0.920, 0.804, and 0.859 eV, are labeled as IX_{1-1} to IX_{1-8}, respectively. Among them, only IX_{1-1} stabilizes after this extended time trace measurement of PL, while others disappear. The IX_{1-1} peak exhibits remarkable air stability over a period of 121 days (Supplementary Fig. 3c), likely due to the chemical inertness of both CNTs and WSe_2.

To gain more insight, we plot the time-trace of PL intensity for each IX (Supplementary Fig. 3d). First, PL from most IXs show abrupt blinking over time, with IX_{1-1} and IX_{1-7} displaying a near "on" and "off" blinking noise. Such pure two-level blinking behavior has been primarily reported in highly confined 0D systems like quantum dots [5] and single molecules [6], suggesting that the interface states here tend to be single emitters. Second, IX_{1-2} and IX_{1-4} correlate with E_{11} when blinking, while other IXs exhibit no clear correlations, implying different origins among the diverse IXs. Third, the blinking of IXs occurs on a scale of tens of seconds, indicative of a slow process. This temporal evolution of PL differs from photobleaching in single molecules originating from chemical reaction [7]. We suspect the observed PL evolution could be related to charge and/or atomic registry. If so, IX_{1-1} could originate from a stable state with a specific configuration, while others represent metastable states that dissipate over time. The atomic reconstruction has been widely observed in 2D-2D heterostructures [8]. In our CNT/WSe_2 samples, the atomic reconstruction is expected to be strong due to the absence of a substrate.

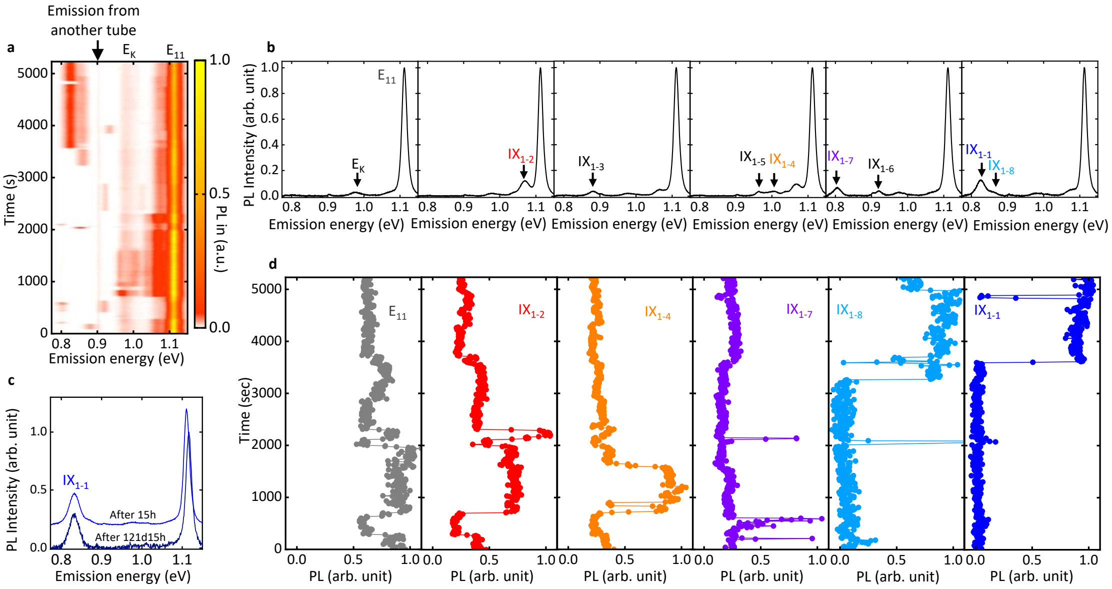

Supplementary Fig. 3 | PL evolution of interface excitons. a, Time-trace PL of the (9,4) CNT/2L WSe_2 sample shown in Fig. 4. A weak emission peak at 0.903 eV comes from the E_{11} emission of an adjacent CNT. **b**, PL spectra at different times showing various IXs. **c**, PL spectra after 15 hours (blue) and after 121 days (black) from the formation of the heterostructure in order to demonstrate a long-scale stability of $IX_{1\text{-}1}$. **d**, Time-trace of the integrated PL intensity for different IX peaks, extracted from **a**. The excitation energy is adjusted to E_{22} of 1.70 eV and the power is 10 μW for (**a**,**b**,**d**) and 5 μW for (**c**).

Supplementary Note 4:
Repeatability of IX peaks in CNT/1L WSe_2 heterostructures

We have prepared three more 1L samples with chirality of (9,4) and (8,6), and performed measurements before and after transfer as shown in Supplementary Fig. 4. After the transfer of 1L WSe_2, the three samples show several red shifted IX peaks, consistent with the 1L sample in Fig. 1.

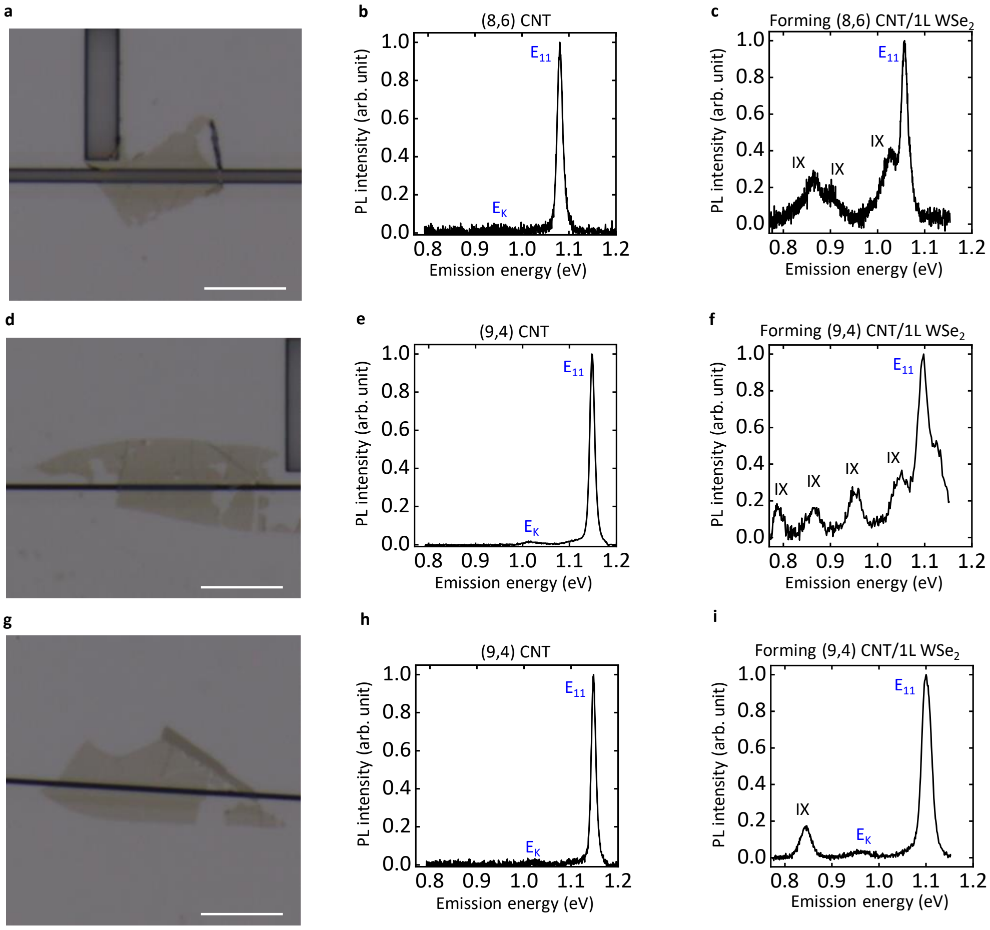

Supplementary Fig. 4 | Other CNT/1L WSe_2 samples with IX peaks. a-c, Optical microscope image (**a**), the PL spectra (**b**) before and (**c**) after forming the heterostructure for the (8,6) CNT/1L WSe_2 sample. The excitation energy is adjusted to E_{22} and the power is 10 μW for (**b**) and 5 μW for (**c**). **d-f,** Optical microscope image (**d**), the PL spectra (**e**) before and (**f**) after forming the heterostructure for the (9,4) CNT/1L WSe_2 sample. The excitation energy is adjusted to E_{22} and the power is 10 μW. **g-i,** Optical microscope image (**g**), the PL spectra (**h**) before and (**i**) after forming the heterostructure for another (9,4) CNT/1L WSe_2 sample. The excitation energy is adjusted to E_{22} and the power is 10 μW for (**h**) and 0.3 μW for (**i**). Scale bars in (**a**,**d**,**g**) are 10 μm.

Supplementary Note 5:
Spatial correlation between interface excitons and E_{11} excitons

In Supplementary Fig. 5, the results of hyperspectral PL imaging from the (9,4) CNT/1L WSe_2 heterostructure in Fig. 1 are summarized. Supplementary Fig. 5a-f show PL spectra at different locations indicated in Supplementary Fig. 5g. P1 is the location of the (9,4) CNT, exhibiting three IX peaks labeled as IX_1, IX_2, and IX_3. Compared to Fig. 1f, one additional IX_3 peak is observed, which is unstable and disappears after this hyperspectral PL imaging measurement. P5 and P6 correspond to spectra from suspended WSe_2, where no IX peaks are observed. If the IX peaks originate from defects in WSe_2, we expect similar emission peaks uniformly distributed over the flake. PL images at the energies of the IX peaks are shown in Supplementary Fig. 5h-j, indicating no other emitters within the image. We note that the images are enlarged because of a strong power saturation behavior of IX peaks (Fig. 3c,d) and a fringe pattern from the optical system.

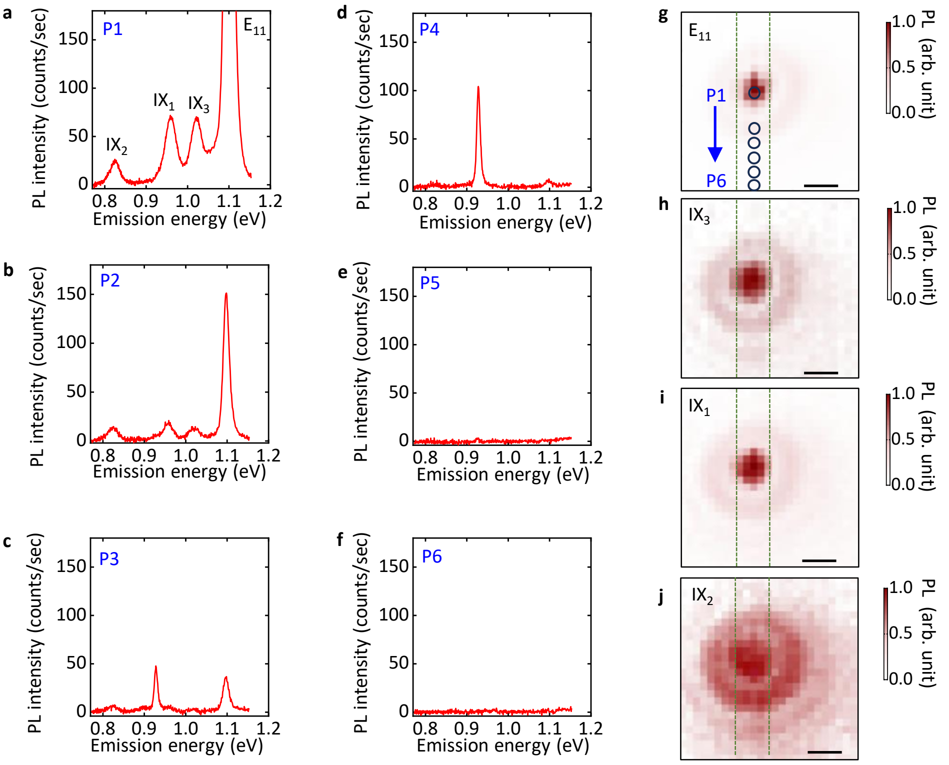

Supplementary Fig. 5 | Hyperspectral PL imaging of IX peaks. a-f, PL spectra taken at six positions, indicated by blue circles as P1, P2, P3, P4, P5, and P6 in (**g**). The sharp peaks at 0.93 eV in spectra from P3 and P4 are from another (9,7) CNT. **g-j**, The PL intensity maps of E_{11}, IX_1, IX_2, and IX_3 for the (9,4) CNT/1L WSe_2 heterostructure as shown in Fig. 1. The scale bars are 1 μm. The apparent sizes of the emission spots differ because of the strong saturation behavior of the interface excitons combined with the Airy pattern. The excitation energy is 1.653 eV and the excitation power is 10 μW.

Supplementary Note 6:
Clarification of the E_{22} excitation peak for (9,4) CNT/WSe_2 heterostructures

The CNT E_{22} excitation peak for the (9,4) CNT/WSe_2 sample in the PLE map of Fig. 1i is energetically close to WSe_2 A exciton absorption (E_A) peak. However, we can distinguish the two peaks from the line shape and the excitation polarization. To demonstrate such differences, we compare a (12,4) CNT/2L WSe_2 sample which is known to exhibit exciton transfer and E_A absorption peak [9] to two (9,4) CNT/2L WSe_2 samples. Supplementary Fig. 6a shows PLE spectra of the E_{11} emission from the three samples. Although the peaks are energetically close, the E_A peak in the (12,4) CNT/2L WSe_2 sample is considerably broader than the peak for the (9,4) CNT/WSe_2 samples.

In addition, excitation polarization measurements of E_{11} emission would result in strong linear polarization for the CNT E_{22} peak, whereas WSe_2 A exciton absorption would be isotropic [9]. As indicated in Supplementary Fig. 6b, the excitation polarization is isotropic for the E_A excitation peak in the (12,4) CNT/2L WSe_2 sample. In comparison, both the (9,4) CNT samples exhibit a strong linear polarization with the degree of 0.84 and 0.61, consistent with the E_{22} excitation peaks. We have measured six heterostructures fabricated from (9,4) CNT, and such polarization dependence has been observed on all of them. Based on such spectral lineshape and polarization dependence observed in multiple samples, we confirm that the peak in Fig. 1i arises from the E_{22} resonance of the (9,4) CNT.

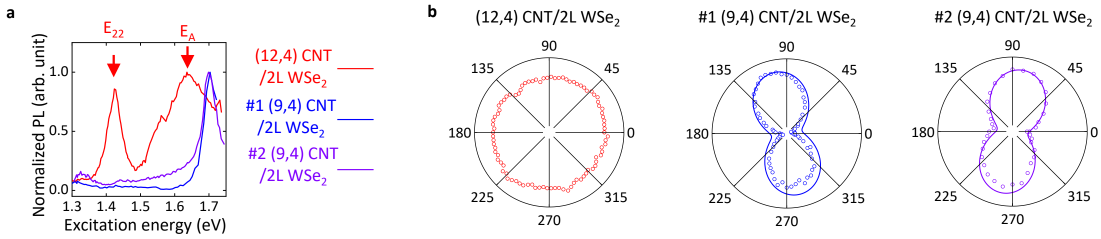

Supplementary Fig. 6 | Differences in E_A and E_{22} excitation peaks. a, Normalized PLE spectra of the E_{11} emission obtained by integrating over a 30-meV-wide spectral window centered at the E_{11} energy from the (12,4) CNT/2L WSe_2 (red) and the two (9,4) CNT/2L WSe_2 samples (blue for #1 and purple for #2). The E_{22} and E_A peaks for the (12,4) CNT/2L WSe_2 sample are labelled by red arrows. The excitation polarization is adjusted along the tube. **b**, Excitation polarization dependence of E_{11} emission for the (12,4) CNT/2L WSe_2 sample (excitation energy: 1.65 eV), the #1 (9,4) CNT/2L WSe_2 sample (excitation energy: 1.69 eV), and the #2 (9,4) CNT/2L WSe_2 sample (excitation energy: 1.70 eV). The lines are fits to a cosine squared function. Excitation power values are 4 μW for the #1 (9,4) CNT/2L WSe_2 sample and 10 μW for the others.

Supplementary Note 7:

Clarification of the absence of IX peaks for heterostructures with small E_{11} energies

To clarify the absence of IX peaks, we have replotted the spectra from several samples in Fig. 2b with a zoomed-in vertical axis as Supplementary Fig. 7. For (14,0) CNT/4L WSe_2, (8,7 CNT)/2L WSe_2, (12,1) CNT/2L WSe_2, and (9,4) CNT/2L WSe_2 heterostructures, the IX peaks can be identified with peak counts larger than 20 counts/sec. For (15,1) CNT/3L WSe_2, (9,8) CNT/1L WSe_2, (12,4) CNT/3L WSe_2, and (13,2) CNT/3L WSe_2 heterostructures, we should be able to detect any peaks higher than 10 counts/sec considering the noise floor. Instead, we do not observe any additional IX peaks, which supports our interpretation with band alignment transition.

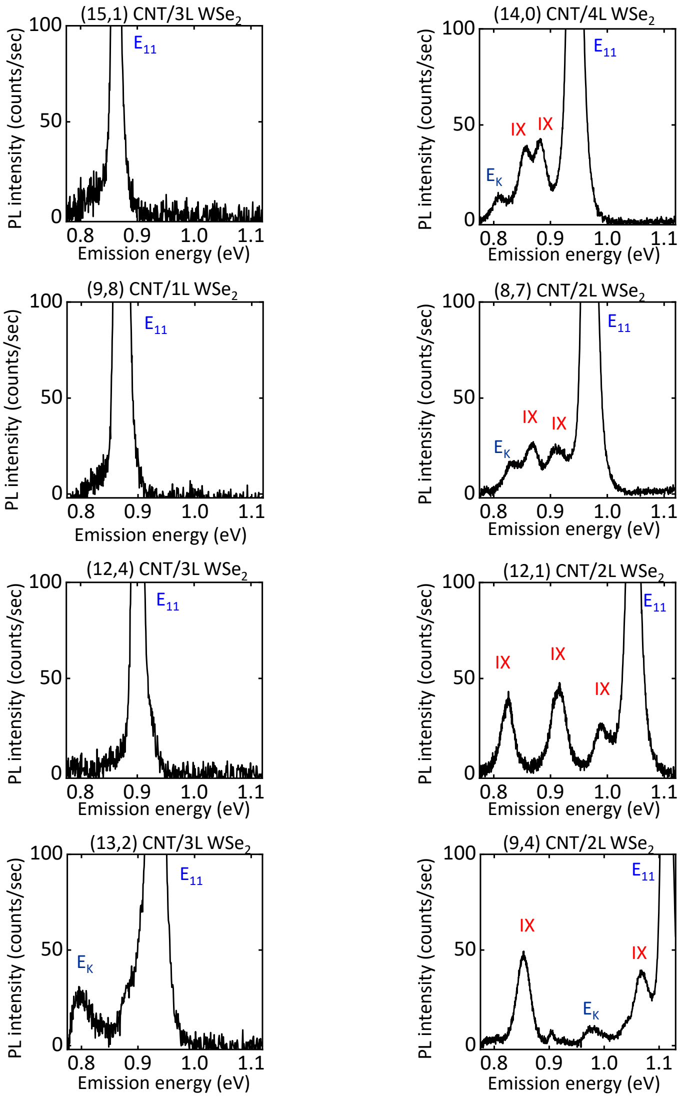

Supplementary Fig. 7 | Zoomed-in PL spectra from different heterostructures. Excitation energy is adjusted to E_{22} for each heterostructure. Excitation power values are 4, 6, and 5 μW for (9,4) CNT/2L WSe_2, (12,1) CNT/2L WSe_2, (8,7) CNT/2L WSe_2 heterostructures, respectively, and 10 μW for other samples.

Supplementary Note 8:
IX peak energies from all the samples

We plot ΔE = E_{11}-E_{IX} versus E_{11} for the IX peaks from all the 35 samples as shown in Supplementary Fig. 8. A black line shows the detector limit. For CNTs exhibiting IX peaks, the highest-energy IX peaks observed are close to the E_{11} energies with ΔE < 0.09 eV, and most of them are located around ΔE ≈ 0.05 eV. The absence of such IX peaks for CNTs with E_{11} < 0.94 eV therefore indicates the interpretation by band alignment transition from type-II to type-I.

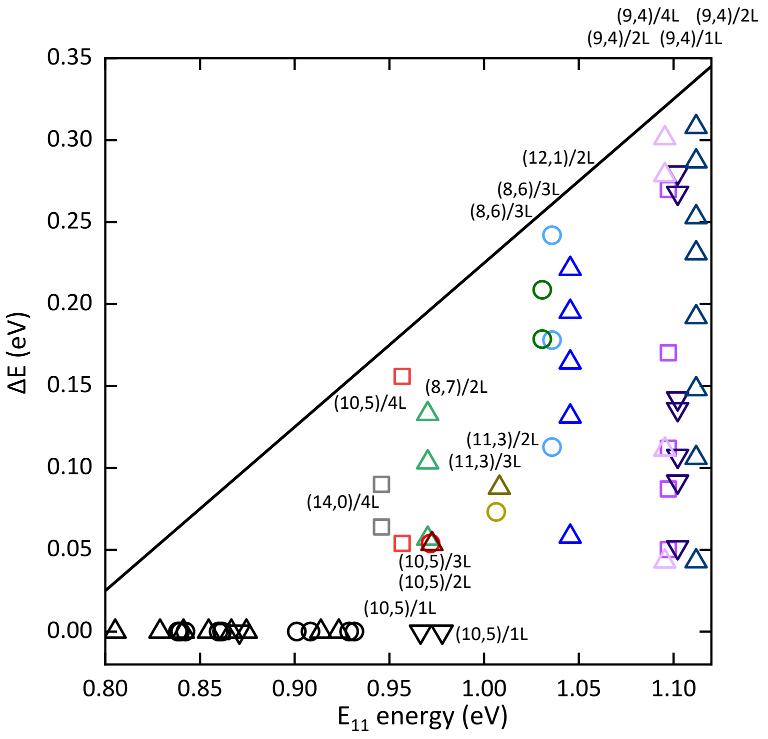

Supplementary Fig. 8 | ΔE plotted as a function of E_{11} energy from 35 samples. The black line indicates the detection limit. Inverted triangles, upright triangles, circles, and rectangles indicate 1L, 2L, 3L, and 4L WSe_2 samples, respectively. For the samples absent of IX peaks, we plot symbols at ΔE = 0 eV.

Supplementary Note 9:

Anticorrelation between the appearance of the IX peaks and exciton transfer process

To clarify the relation between the appearance of the IX peaks and exciton transfer process, we present more PLE maps from different samples as shown in Supplementary Fig. 9. (12,1) and (14,0) CNT based heterostructures do not show the E_A excitation peak, while the new low-energy emission peaks are observed. In comparison, the other CNTs with smaller bandgap exhibit exciton transfer and the IX peaks could not be resolved. Such anticorrelation is consistent with the band alignment transition as exciton transfer mainly occurs for type-I band alignment.

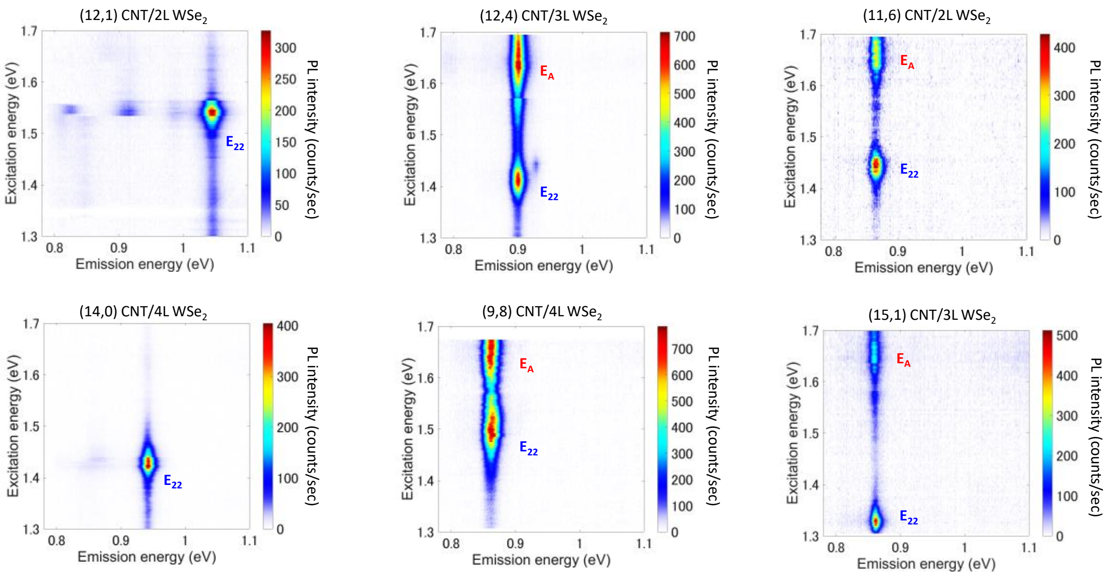

Supplementary Fig. 9 | PLE maps from different heterostructures. The excitation power is 5 μW for (12,1) CNT/2L WSe_2 and (14,0) CNT/4L WSe_2 samples and 10 μW for others. The excitation polarization is aligned to CNT axis.

Supplementary Note 10:
Consideration of holes in WSe_2 to account for band alignment transitions

In our type-II heterostructures, electrons should reside within CNTs while holes should be located in WSe_2. The valence band maxima (VBM) for WSe_2 are therefore of our interest, and their energies at the K and Γ points are extracted from the DFT simulations and plotted in Supplementary Fig. 10a. The energy is higher at the K point for 1L and at the Γ point for thicker ones, resulting in the well-known transition from direct gap to indirect gap. The Γ point shows a substantial shift of 0.515 eV from 1L to 2L, whereas the K point shows a modest increase by 0.036 eV.

Experimentally, we compare 1L and 2L WSe_2 for four distinct CNT chiralities (9,4), (8,6), (10,5), (9,8) as shown in Supplementary Fig. 10b. In (9,4) and (8,6) heterostructures, IX peaks are observed for both 1L and 2L with no apparent layer number dependence, indicating a full type-II band alignment. (10,5) heterostructures exhibit a layer number dependence as also shown in Fig. 2d. Only the 2L shows the IX peak, implying a transition from type-II to type-I. In (9,8) heterostructures, IX peaks are absent for both 1L and 2L, consistent with type-I band alignment.

If we assume that the electronic states at the Γ point play an important role, we expect to see band alignment transitions in many chiralities as the VBM at the Γ point shows a large change in energy. For 2L samples, we observe band alignment transition from type-I to type-II as the E_{11} energy is increased by 0.105 eV from (9,8) to (10,5). For (8,6) and (9,4), the E_{11} energy further increases by 0.065 and 0.142 eV, respectively, and the valence band energy reduction should be within this energy range. In comparison, the VBM energy at the Γ point decreases by more than 0.5 eV for 1L compared to 2L. This change in VBM is much greater than the difference in the E_{11} energy, and therefore we expect the band alignment transitions back to type-I when we replace 2L WSe_2 with 1L WSe_2 in (10,5), (8,6), and (9,4). Experimental results show that the transition is only observed for (10,5), in contradiction with the assumption.

A more reasonable interpretation is that the states at the K point is important for the formation of the interface excitons. The VBM at K point shows a mere 0.036 eV change from 1L to 2L, and is consistent with the fact that we only observe band alignment transition for (10,5). This interpretation is also supported by the appearance of the IX peaks anticorrelated with exciton transfer. The A exciton in WSe_2 plays an important role in the exciton transfer as reported in [9], which is formed by electron and hole states at the K point. In this case, the band alignment with the states at the K point should determine whether exciton transfer is allowed. Because the IX peaks are anticorrelated with exciton transfer, it is implied that formation of interface excitons is also determined by band alignment of the hole states at the K point.

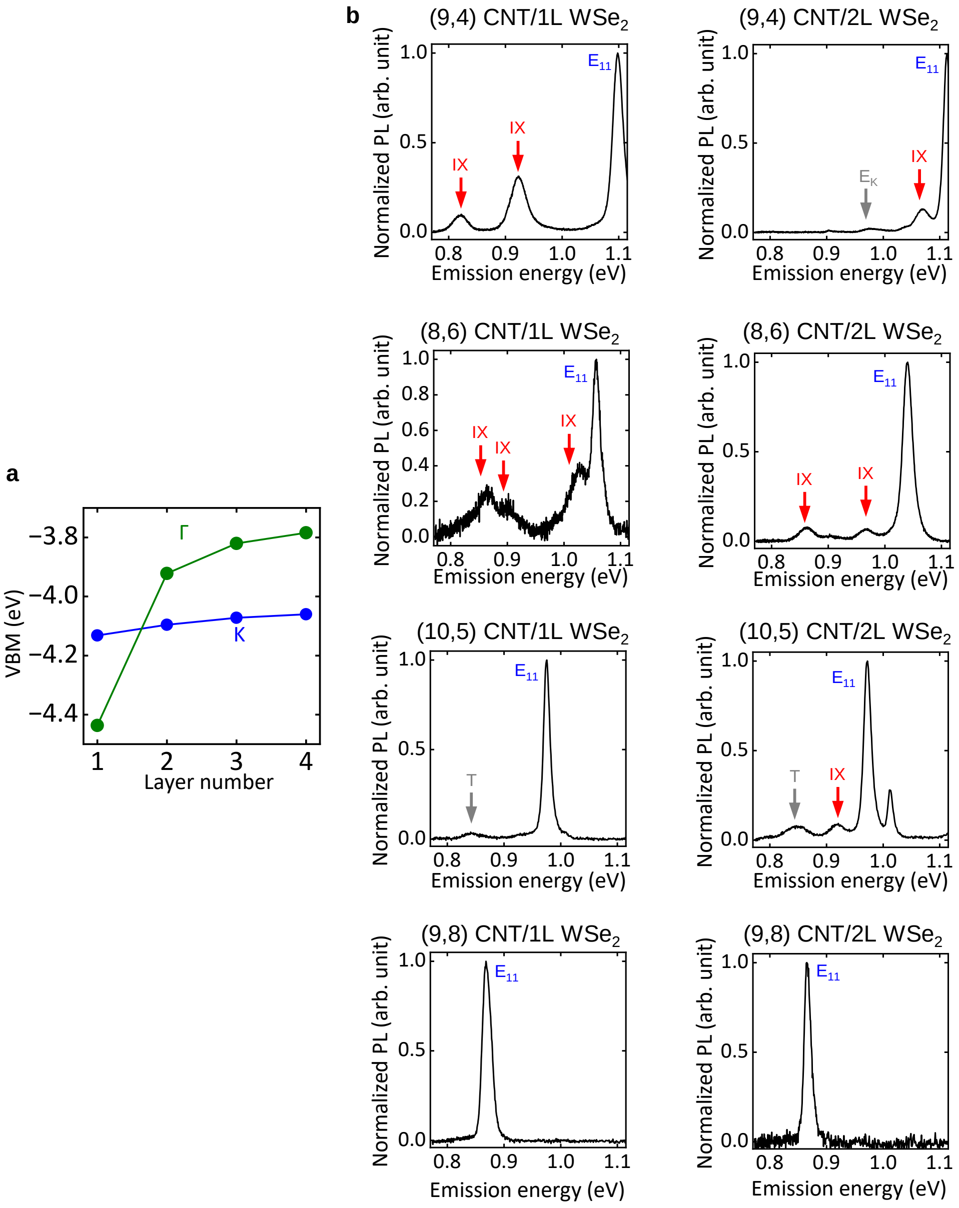

Supplementary Fig. 10 | DFT simulation and the band alignment transition in 1L and 2L WSe_2 samples. a, Calculated VBM energies as a function of WSe_2 layer number. The energies are measured from the vacuum level. **b**, The normalized PL spectra for the (9,4), (8,6), (10,5), (9,8) CNT/1L and 2L WSe_2 heterostructures, respectively. The excitation energies are adjusted to E_{22} energies. A weak emission peak at 1.013 eV in (10,5) CNT/2L WSe_2 sample comes from the E_{11} emission of an adjacent CNT.

Supplementary Note 11:
Emission polarization angle in other samples

The emission polarization from the (9,4) CNT/1L WSe_2 sample (Fig. 3a) reveals an angle difference between the E_{11} exciton and the interface excitons. Here we focus on (9,4) CNT/2L WSe_2 heterostructures to further investigate such deviation. Supplementary Fig. 11a is taken from the sample shown in Supplementary Fig. 3, and the polarization angle of IX_{1-1} has a slight deviation from that of E_{11} by 7.1°. Another prepared (9,4) CNT/2L WSe_2 sample exhibits three interface excitons at energies of 1.056, 0.823, and 0.801 eV (Supplementary Fig. 12a,b), and are labeled as IX_{2-1}, IX_{2-2}, and IX_{2-3}, respectively. The polarization angle of IX_{2-1}, IX_{2-2}, and IX_{2-3} clearly deviates from that of E_{11}, with values of 21.0°, 17.8°, and 20.4°, respectively. Such variations in the polarization angle cannot be solely explained by artifacts of the optical system, but suggesting the distinct distortions of interfacial dipoles in different samples.

We speculate that the linear polarization observed in interface excitons arises from the one-dimensional nature of electrons within CNTs. The band alignment studies in Fig. 2 suggest that electrons are predominantly within CNTs while holes are located in WSe_2. Given the inherent 1D nature of electrons in CNTs, the movement of electrons is constrained along the 1D axis. In such mixed-dimensional systems, a complex scenario of tilted dipoles involving both vertical and in-plane components may be able to explain the experimental observations. If the tilting of the dipole is caused by the structural anisotropy and/or strain, the direction of the tilt is not necessarily along the nanotube axis, which possibly explains the deviations in polarization angle.

In another prepared (9,4) CNT/2L WSe_2 sample, we observed an intriguing IX peak with more drastic polarization deviation, which is labeled as IX_{8-2} in Supplementary Fig. 11c,d. Distinct from other observed IX peaks such as IX_{8-1} and IXR_{8-3}, IX_{8-2} displays an almost perpendicular angle misalignment with the E_{11} exciton. Moreover, we observed a remarkably lower in-plane linear polarization degree of 0.41 compared with other IX peaks.

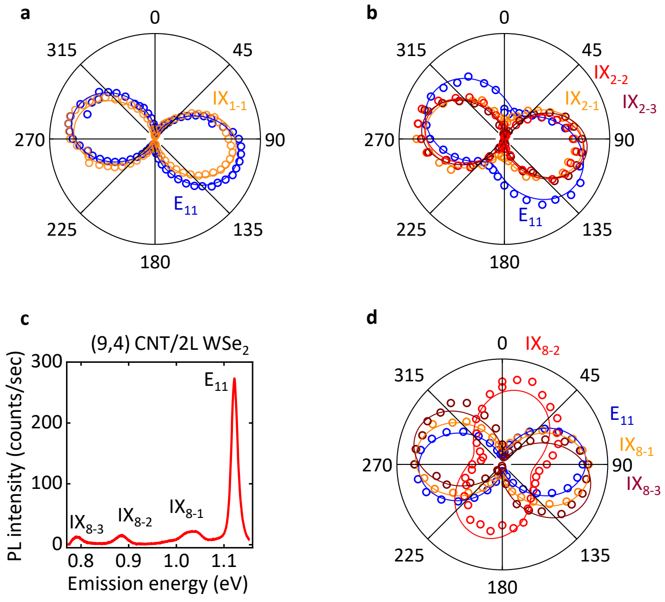

Supplementary Fig. 11 | Emission polarization measurements in (9,4) CNT/2L WSe_2 samples. a, Emission polarization dependence of PL emission from E_{11} (blue circles) and IX_{1-1} (orange circles). The lines are fits to a cosine squared function. The excitation energy is 1.699 eV and excitation power is 4 μW. **b**, Emission polarization dependence of PL emission from E_{11} (blue circles), IX_{2-1} (orange circles), IX_{2-2} (red circles), and IX_{2-3} (wine circles). The lines are fits to a cosine squared function. The excitation energy is 1.669 eV and excitation power is 1 μW. **c**,**d** A PL spectrum from the (9,4) CNT/2L WSe_2 heterostructure (**c**) and emission polarization dependence of PL emission (**d**) from E_{11} (blue circles), IX_{8-1} (orange circles), IX_{8-2} (red circles), and IX_{8-3} (wine circles). The lines are fits to a cosine squared function. The excitation energy is 1.703 eV and excitation power is 10 μW.

Supplementary Note 12:
Laser power saturation behaviors in IXs

The IXs shown in Fig. 3 are strongly confined, as characterized by the power dependence of the PL spectra. In other samples where interface excitons are also observed, we note that the saturation behavior depends on the energy difference between E_{11} and interface excitons.

PL spectra taken from the (9,4)/2L WSe_2 sample at powers of 0.2 μW and 2 μW are shown in Supplementary Fig. 12a,b. Power dependence of the integrated PL for each peak is plotted in Supplementary Fig. 12c. The E_{11} peak located at 1.099 eV exhibits quasi-linear power dependence. The $IX_{2\text{-}1}$ peak, with energy close to E_{11}, shows similar power dependence. In contrast, a clear saturation behavior is observed for the low-energy $IX_{2\text{-}2}$ and $IX_{2\text{-}3}$ peaks. Such a peak-energy dependent saturation behavior can be caused by the different trap potentials in IXs, and in which case $IX_{2\text{-}2}$ and $IX_{2\text{-}3}$ would have large potentials that result in strong confinement. In comparison, $IX_{2\text{-}1}$ could be weakly confined due to a shallow potential.

Similar energy-dependent power saturation is also confirmed in other samples, as shown in Supplementary Fig. 12d-r. Low-energy $IX_{1\text{-}1}$, $IX_{3\text{-}2}$, and $IX_{3\text{-}3}$ peaks show substantial saturation behavior. In contrast, high-energy peaks near E_{11} such as $IX_{4\text{-}1}$, $IX_{5\text{-}1}$, $IX_{6\text{-}1}$, and $IX_{6\text{-}2}$ exhibit similar power dependence with E_{11} and E_K. These IXs could be highly delocalized, and we do not expect quantum emission from these states (see Supplementary Fig. 15).

Supplementary information

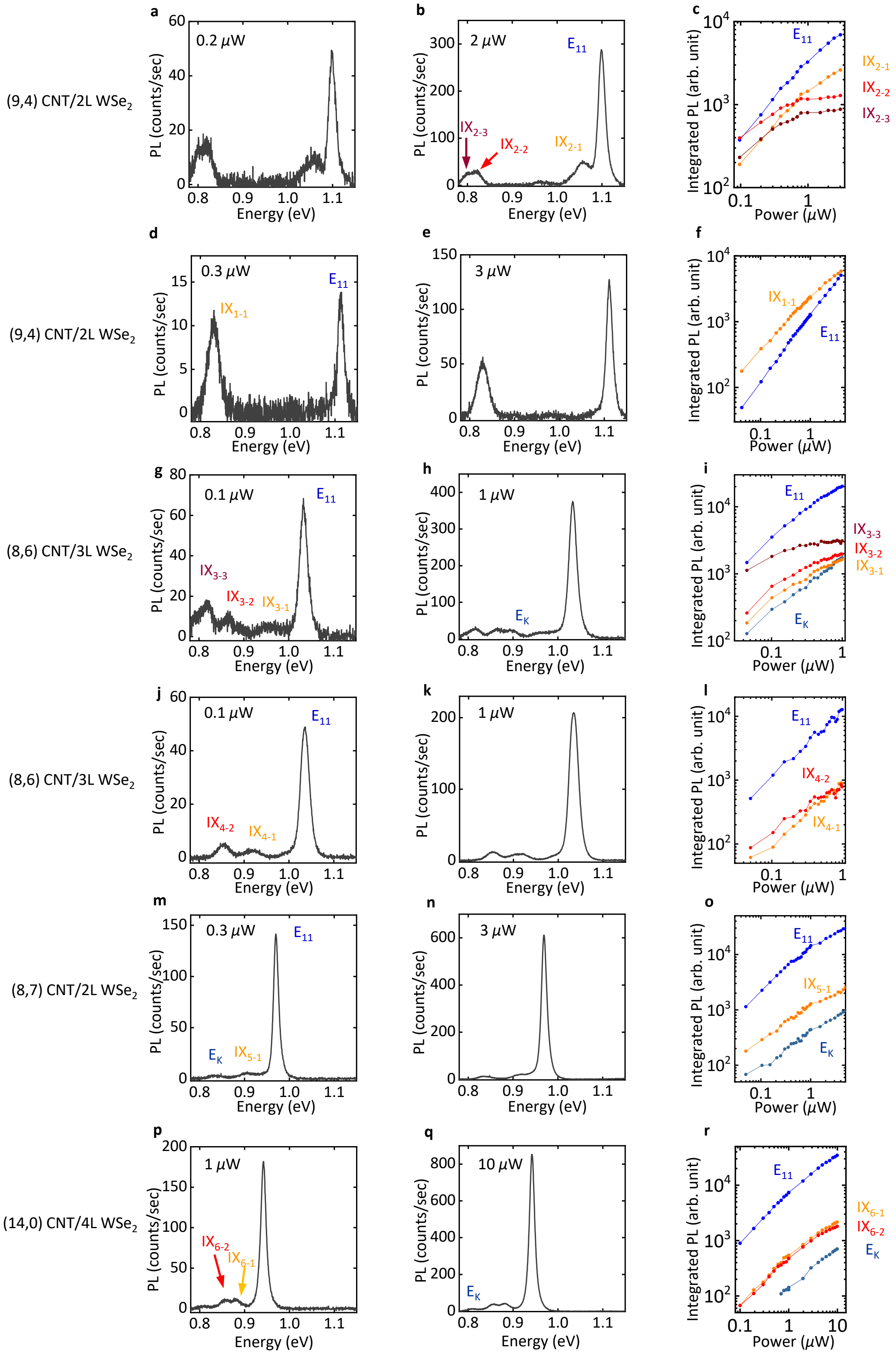

Supplementary Fig. 12 | Laser power dependence of IXs in different samples. a-c, PL spectra for the (9,4) CNT/2L WSe_2 sample shown in Supplementary Fig. 11b at powers of 0.2 μW (**a**) and 2 μW (**b**). (**c**) The laser power dependence of the integrated intensity for the different PL peaks in (**a**,**b**). **d-f,** PL spectra for the (9,4) CNT/2L WSe_2 sample shown in Supplementary Fig. 3 at powers of 0.3 μW (**d**) and 3 μW (**e**). (**f**) The laser power dependence of the integrated intensity for the different PL peaks in (**d**,**e**). **g-i,** PL spectra for the (8,6) CNT/3L WSe_2 sample shown in Fig. 2b at powers of 0.1 μW (**g**) and 1 μW (**h**). (**i**) The laser power dependence of the integrated intensity for the different PL peaks in (**g**,**h**). **j-l,** PL spectra for another (8,6) CNT/3L WSe_2 sample at powers of 0.1 μW (**j**) and 1 μW (**k**). (**l**) The laser power dependence of the integrated intensity for the different PL peaks in (**j**,**k**). **m-o,** PL spectra for the (8,7) CNT/2L WSe_2 sample shown in Fig. 2b at powers of 0.3 μW (**m**) and 3 μW (**n**). (**o**) The laser power dependence of the integrated intensity for the different PL peaks in (**m**,**n**). **p-r,** PL spectra for the (14,0) CNT/4L WSe_2 sample shown in Fig. 2b at powers of 1 μW (**p**) and 10 μW (**q**). (**r**) The laser power dependence of the integrated intensity for the different PL peaks in (**p**,**q**). Excitation energy is adjusted to E_{22} for each heterostructure.

Supplementary Note 13:
Abrupt blinking noise from IXs

Most low-energy peaks show near “on” and “off” two-state blinking noise. Here we demonstrate the long time scale PL trace of an IX peak from another prepared (9,4) CNT/2L WSe_2 sample, which exhibits several transitions. The unstable IX peak is indicated in Supplementary Fig. 13a. The PL trace from this peak is shown in Supplementary Fig. 13b. The PL intensity shows clear two levels and blinking over a long time scale of 20,000 s.

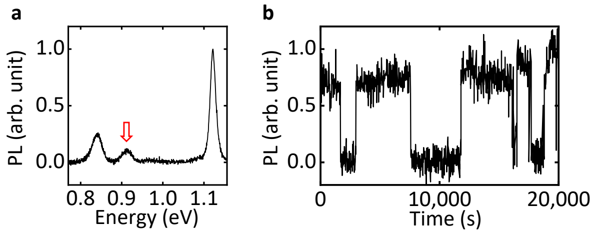

Supplementary Fig. 13 | Long time scale PL trace of an IX peak. a, A PL spectrum for another (9,4) CNT/2L WSe_2 sample. **b**, Time-trace of the IX peak as indicated by a red arrow in (**a**). The excitation energy is adjusted to 1.703 eV of E_{22} and the excitation power is 5 μW.

Supplementary Note 14:
Background correction for second-order photon correlation results

In order to determine the intrinsic $g^{(2)}(0)$ value for interface excitons, we need to consider the effects from the uncorrelated background PL. Supplementary Fig. 14a displays the PL spectrum from the (9,4) CNT/2L WSe_2 heterostructure, on which photon correlation measurements in Fig. 4 are conducted. In the photon correlation analysis, we subtract the background signal as indicated in the PL spectrum. Supplementary Fig. 14b presents the raw number of coincidences. The $g^{(2)}(\tau)$ shown in Fig. 4 is calculated by $g^{(2)}(\tau) = (g^{(2)}_{raw} - 1)/\rho^2 + 1$ where $g^{(2)}_{raw}$ is the normalized uncorrected correlation function [10]. Here, ρ = 0.89 represents the ratio between the $IX_{1\text{-}1}$ PL intensity and the total integrated PL intensity including the background signal.

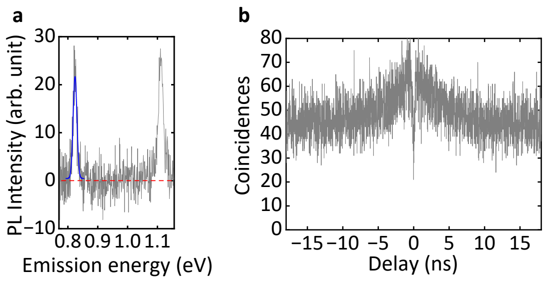

Supplementary Fig. 14 | Raw second-order correlation results. a, A PL spectrum from the (9,4) CNT/2L WSe_2 heterostructure (Supplementary Fig. 3). Peaks at 0.825 and 1.112 eV correspond to $IX_{1\text{-}1}$ and E_{11}, respectively. The grey line represents experimental results, while the blue line is a Gaussian fit for the $IX_{1\text{-}1}$ peak. The red dashed line indicates the 0 count level, illustrating the presence of an uncorrelated background. **b**, The raw number of coincidences in a second-order correlation measurement for the $IX_{1\text{-}1}$ PL. A longpass filter (0.886 eV) is employed to exclude PL signals from E_{11}.

Supplementary Note 15:
Second-order correlation statistics of other IX peaks

We also carry out second-order photon correlation measurements for other heterostructures. Supplementary Fig. 15a shows a PL spectrum from an (12,0) CNT/2L WSe_2 heterostructure, where a stable low-energy interface exciton peak $IX_{7\text{-}1}$ is observed at 0.811 eV. The second-order photon correlation measurement for this peak displays a clear antibunching dip along with a bunching peak. Moreover, the value of $g^{(2)}(0) = 0.33$ suggests single-photon emission from this interface exciton. In the case of the (9,4) CNT/2L WSe_2 sample used in Supplementary Fig. 12a,b, $IX_{2\text{-}2}$ and $IX_{2\text{-}3}$ are energetically so close to each other that they cannot be separately filtered. We thus measure the correlation of the PL emission from both peaks. The statistics are shown in Supplementary Fig. 15c, and also shows an antibunching dip and a bunching peak. Compared with Fig. 4a and Supplementary Fig. 15b, the antibunching behavior here is less pronounced with $g^{(2)}(0) = 0.61$, which could be explained by the coexistence of two single-photon sources. Additionally, we conduct correlation measurements of PL emission from $IX_{6\text{-}1}$ and $IX_{6\text{-}2}$ in the (14,0) CNT/4L WSe_2 sample shown in Supplementary Fig. 12p,q, and neither antibunching nor bunching behavior is observed (Supplementary Fig. 15d). It is consistent with the quasi-linear power dependence of these high-energy peaks, suggesting that they are free excitons rather than trapped ones.

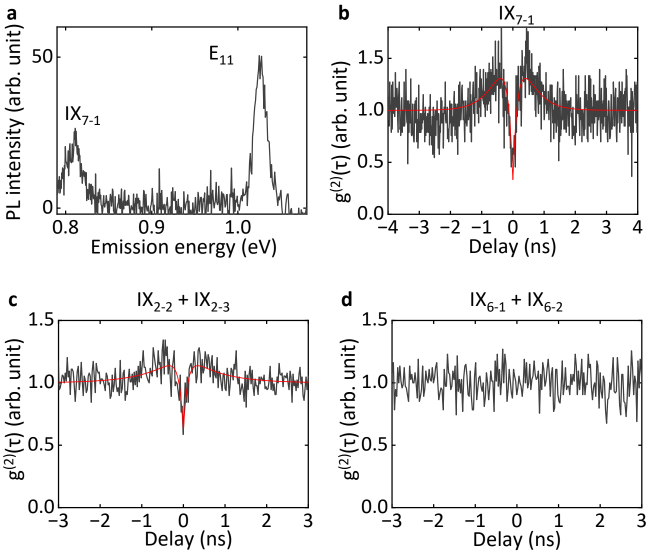

Supplementary Fig. 15 | Second-order correlation statistics of confined and free interface excitons. a, A PL spectrum from the (12,0) CNT/2L WSe_2 heterostructure. Peaks at 0.811 and 1.026 eV correspond to $IX_{7\text{-}1}$ and E_{11}, respectively. **b**, Second-order correlation statistics of $IX_{7\text{-}1}$. The excitation energy is adjusted to 1.531 eV of E_{22} with a continuous-wave laser power of 0.5 μW. A longpass filter (0.826 eV) is used to collect PL emission from $IX_{7\text{-}1}$. From the fitting, we extract τ_A and τ_B as 0.295 and 0.406 ns, and

α and β as 0.89 and 0.30, respectively. **c**, Second-order correlation statistics of $IX_{2\text{-}2}$ and $IX_{2\text{-}3}$ from the (9,4) CNT/2L WSe_2 sample. The excitation energy is adjusted to 1.664 eV of E_{22} with a continuous-wave laser power of 0.4 μW. A longpass filter (0.855 eV) is used to collect PL emission from $IX_{2\text{-}2}$ and $IX_{2\text{-}3}$. From the fitting, we extract τ_A and τ_B as 0.104 and 0.757 ns, and α and β as 0.51 and 0.25, respectively. **d**, Second-order correlation statistics of $IX_{6\text{-}1}$ and $IX_{6\text{-}2}$ from the (14,0) CNT/4L WSe_2 sample. The excitation energy is adjusted to 1.435 eV of E_{22} with a continuous-wave laser power of 5 μW. A longpass filter (0.886 eV) is used to collect PL emission from $IX_{6\text{-}1}$ and $IX_{6\text{-}2}$. The grey lines are experimental results, and the red line is the fitting. The second-correlation statistics data are binned to reduce the noise. Since the background PL is small for these three samples, we do not perform background correction for the second-order correlation data.

Supplementary Note 16:
Second-order correlation statistics at low laser power

We have conducted additional second-order photon correlation measurement with the (9,4) CNT/1L WSe_2 sample in Supplementary Fig. 4i, where a stable IX peak is observed. The photon correlation results from the IX peak without any background correction are presented in Supplementary Fig. 16 under both linear and logarithmic scales [11]. It is confirmed that the photon correlation function is normalized to 1 up to dozens of nanoseconds. Notably, the sample exhibits reduced bunching behavior and a rapid antibunching timescale of 0.133 ns, which may be attributed to the use of low laser power. The fitted value of $g^{(2)}(0) = 0.31$ again suggests the single-photon emission from this interface exciton.

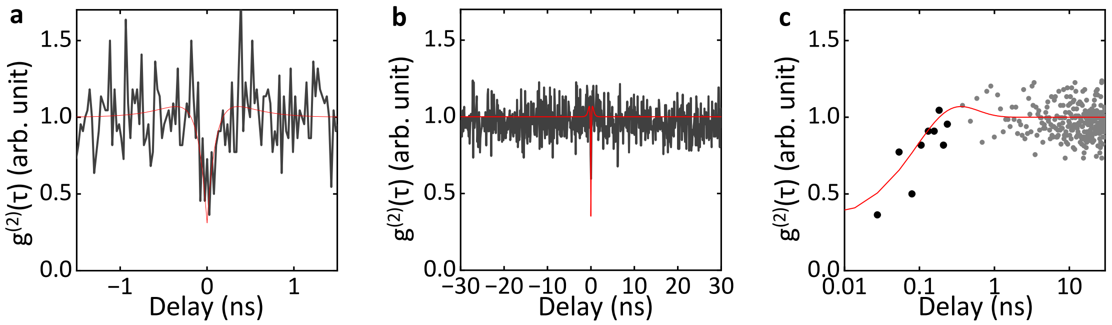

Supplementary Fig. 16 | Second-order correlation statistics with reduced bunching behavior. a, The second-order correlation statistics for the IX peak shown in Supplementary Fig. 4i. A longpass filter (0.919 eV) is employed to exclude PL signals from E_{11}. The excitation energy is adjusted to E_{22} of 1.699 eV and a power of 0.15 μW. The red line is a fitting. From the fitting, we extract τ_A and τ_B as 0.133 and 0.324 ns, and α and β as 0.79 and 0.42, respectively. **b**,**c** The long-time second-order correlation statistics for the samples under (**b**) linear and (**c**) logarithmic scales. The black line for the linear scale and the dots for the logarithmic scale are experimental results. The red lines are fits. Four data points are binned together for the linear scale data and the gray dots.

Supplementary Note 17:
Morphology of suspended CNT/WSe_2 heterostructures

The morphology of suspended CNT/WSe_2 heterostructures have been characterized by using atomic force microscope (AFM), as illustrated in Supplementary Fig. 17. 2D and 3D AFM images in Supplementary Fig. 17b,c are taken at the suspended region, which is marked in Supplementary Fig. 17a. A rough surface is characterized above the substrate, which is attributed to multiple CNT tubes and Fe catalyst. On the other hand, minimal surface roughness is observed for the suspended part of the WSe_2 flake, highlighting the cleanliness of our transfer process. The suspended section falls into the trench by approximately 6 nm, resulting in an intimate contact with the CNTs. Two CNTs are discernible across the trench, as marked by arrows in Supplementary Fig. 17b. The height of the upper CNT is 1.05 nm, which is consistent with the CNT diameter discussed in this paper. In the upper CNT, we also observe a shallow local dip with a depth of ~3 nm and a width of ~350 nm, which may confine interface excitons nearby.

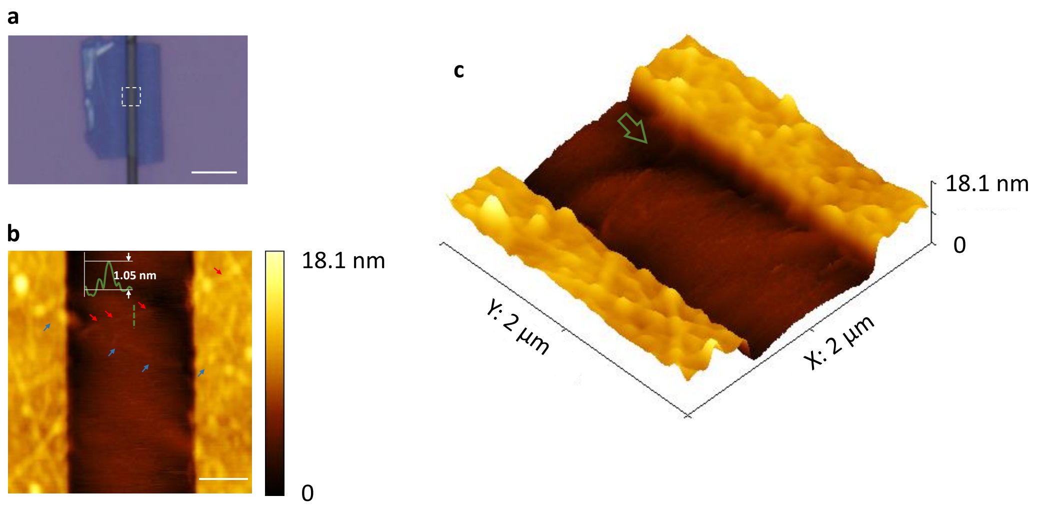

Supplementary Fig. 17 | Identifying CNTs and local strain in the heterostructure. a, An optical microscope image of CNT/2L WSe_2 heterostructures. 2D (**b**) and 3D (**c**) AFM images for the area indicated by the white broken rectangle in (**a**). The inset in (**b**) is a line profile indicated by the green broken line. Red and blue arrows in (**b**) indicate two tubes, while the green arrow in (**c**) indicate the local strain. The scale bars are 5 and 0.4 μm for (**a**) and (**b**), respectively.

Supplementary information

Sample	CNT chirality	WSe_2 layer number	E_{11} (eV)	*N*
#1	(9,4)	2	1.115	8
#2	(9,4)	1	1.102	7
#3	(9,4)	4	1.097	5
#4	(9,4)	2	1.095	4
#5	(12,1)	2	1.046	5
#6	(8,6)	3	1.036	3
#7	(8,6)	3	1.031	2
#8	(11,3)	2	1.008	1
#9	(11,3)	3	1.006	1
#10	(10,5)	1	0.978	0
#11	(10,5)	2	0.973	1
#12	(10,5)	3	0.972	1
#13	(8,7)	2	0.970	2
#14	(10,5)	1	0.966	0
#15	(10,5)	4	0.957	2
#16	(14,0)	4	0.946	2
#17	(14,0)	3	0.932	0
#18	(13,2)	3	0.929	0
#19	(12,4)	2	0.923	0
#20	(9,7)	2	0.914	0
#21	(9,7)	3	0.908	0
#22	(12,4)	3	0.901	0
#23	(11,6)	2	0.874	0
#24	(9,8)	1	0.871	0
#25	(11,6)	2	0.867	0
#26	(15,1)	3	0.862	0
#27	(9,8)	4	0.862	0
#28	(9,8)	3	0.860	0
#29	(9,8)	2	0.855	0
#30	(10,8)	3	0.842	0
#31	(13,5)	2	0.841	0
#32	(14,3)	3	0.840	0
#33	(10,8)	3	0.838	0
#34	(10,8)	2	0.829	0
#35	(12,5)	2	0.805	0

Supplementary Table 1 | The number of the IX peaks *N* for all the samples.

Supplementary References

1. Torrens, O. N., Zheng, M. & Kikkawa, J. M. Energy of K-momentum dark excitons in carbon nanotubes by optical spectroscopy. *Phys. Rev. Lett.* **101**, 157401 (2008).

2. Vora, P. M., Tu, X., Mele, E. J., Zheng, M. & Kikkawa, J. M. Chirality dependence of the K-momentum dark excitons in carbon nanotubes. *Phys. Rev. B*, **81**, 155123 (2010).

3. Blackburn, J. L., Holt, J. M., Irurzun, V. M., Resasco, D. E. & Rumbles, G. Confirmation of K-momentum dark exciton vibronic sidebands using 13C-labeled, highly enriched (6, 5) single-walled carbon nanotubes. *Nano Lett.* **12**, 1398 (2012).

4. Zhao, W., Ghorannevis, Z., Chu, L., Toh, M., Kloc, C., Tan, P.H. & Eda, G. Evolution of electronic structure in atomically thin sheets of WS_2 and WSe_2. *ACS Nano* **7**, 791 (2013).

5. Frantsuzov, P., Kuno, M., Janko, B. & Marcus, R. A. Universal emission intermittency in quantum dots, nanorods and nanowires. *Nat. Phys.* **4**, 519 (2008).

6. Moerner, W. E. & Orrit, M. Illuminating single molecules in condensed matter. *Science* **283**, 1670 (1999).

7. Gordon, M. P., Ha, T. & Selvin, P. R. Single-molecule high-resolution imaging with photobleaching. *Proc. Natl. Acad. Sci.* **101**, 6462 (2004).

8. Weston, A., Zou, Y., Enaldiev, V., Summerfield, A., Clark, N., Zólyomi, V., Graham, A., Yelgel, C., Magorrian, S., Zhou, M., Zultak, J., Hopkinson, D., Barinov, A., Bointon, T. H., Kretinin, A., Wilson, N. R., Beton, P. H., Fal'ko, V. I., Haigh, S. J. & Gorbachev, R. Atomic reconstruction in twisted bilayers of transition metal dichalcogenides. *Nat. Nanotechnol.* **15**, 592-597 (2020).

9. Fang, N., Yamashita, D., Fujii, S., Maruyama, M., Gao, Y., Chang, Y.-R., Fong, C. F., Otsuka, K., Nagashio, K., Okada, S. & Kato, Y. K. Resonant exciton transfer in mixed-dimensional heterostructures for overcoming dimensional restrictions in optical processes. *Nat. Commun.* **14**, 8152 (2023).

10. Brouri, R., Beveratos, A., Poizat, J. P. & Grangier, P. Photon antibunching in the fluorescence of individual color centers in diamond. *Opt. Lett.* **25**, 1294 (2000).

11. Patel R. N., Hopper D. A., Gusdorff J. A., Turiansky M. E., Huang T.-Y., Fishman R. E. K., Porat B., Walle C. G. V. d. & Bassett L. C. Probing the optical dynamics of quantum emitters in hexagonal boron nitride. *PRX Quantum* **3**, 030331 (2022).
